# Conventional Penile Reconstruction Versus Penile Allotransplantation: A Comprehensive Review

**DOI:** 10.7759/cureus.94801

**Published:** 2025-10-17

**Authors:** Naga Anvesh Kodali, Ramu Janarthanan, Omer Faruk Dirican, Zeynep Demir, Bedreddin Sazoglu, Vijay S Gorantla, Yalcin Kulahci, Fatih Zor

**Affiliations:** 1 Surgery, Wake Forest School of Medicine, Winston-Salem, USA; 2 Plastic and Reconstructive Surgery, Amrita Institute of Medical Sciences and Research Centre, Amrita Vishwa Vidyapeetham, Kochi, IND; 3 Plastic and Reconstructive Surgery, Indiana University School of Medicine, Indianapolis, USA

**Keywords:** microsurgical reconstruction, penile allotransplantation, penile reconstruction, vascularized composite allotransplantation, vca

## Abstract

Penile loss from trauma, oncologic resection, congenital anomalies, or gender-affirming needs represents a devastating condition with profound functional and psychosocial implications. Conventional reconstructive techniques, including radial forearm, fibula, scapular, and anterolateral thigh flaps, have evolved over decades and provide patients with an acceptable neophallus capable of voiding and sexual activity. However, these methods remain limited by donor-site morbidity, prosthesis-related complications, high revision rates, and the inability to restore natural erectile tissue or fully replicate penile appearance. In recent years, penile allotransplantation has emerged as an alternative within the field of vascularized composite allotransplantation, offering restoration of native anatomy and the potential to achieve sensation, erectile function, and even fertility. To date, only five penile transplantations have been reported worldwide, each demonstrating encouraging short-term outcomes but raising complex challenges. The primary concerns are the lifelong requirement for systemic immunosuppression, the unknown long-term consequences of rejection episodes, and significant ethical and psychosocial considerations, including donor allocation, patient selection, and recipient and partner acceptance. In this narrative review, we compared conventional reconstructive approaches with transplantation, drawing on published literature and clinical experience to highlight indications, surgical techniques, functional outcomes, and psychosocial dimensions. While reconstruction remains the standard of care and provides reliable outcomes compatible with normal life expectancy, transplantation holds promise as a quality-of-life-enhancing option for carefully selected patients. Ongoing clinical research, refinement of surgical and immunological strategies, and the development of robust preclinical models will be essential in defining the role of penile allotransplantation in the future.

## Introduction and background

The loss of the penis induces notable physical and psychosocial distress among men. Research has demonstrated that penile reconstruction significantly influences the quality of life of affected individuals [1-6]. The causes of penile loss can stem from oncologic surgical resection, trauma, or congenital conditions. Another demographic seeking penile reconstruction includes transgender patients [7-12].

The initial penile reconstruction procedure was conducted in 1936 by Nikolaj Bogoraz [13-15], a Russian surgeon, utilizing a tubed lower abdominal flap with rib cartilage to impart stiffness for potential sexual activity. However, this approach lacked efforts towards urethral reconstruction or ensuring sufficient protective and erogenous sensation. Subsequently, in 1948, Gilles and Harrison [16] expanded upon this technique, introducing the contemporary "tube within a tube" design for neourethra creation. Nevertheless, this reconstruction method necessitated three or more stages and produced outcomes that varied widely.

Prior to the microsurgery era, lower abdominal flaps, including the groin flap, remained a common method for penile reconstruction. Subsequently, various techniques employing microsurgical methods were introduced for the purpose of penile reconstruction [13,14,17,18]. The objectives of penile reconstruction were outlined by Hage et al. in 1993 [8,19]. Ideally, the procedure should be completed in a single stage and result in the formation of a functional urethra enabling standing urination [11,13,14,20,21]. Furthermore, achieving tactile and erogenous sensation, rigidity, adequate volume, minimal complications at the donor site, and a visually natural appearance of the penis are additional criteria for successful penile reconstruction. Recently, penile transplantation became incorporated into the repertoire of techniques for penile reconstruction [4,22,23].

However, none of the techniques described in the existing literature fully satisfy these objectives [24]. Here, we provide an overview of the traditional approaches to penile reconstruction and contrast these methods with penile transplantation with regard to the goals of restoration of penile function. Furthermore, we examine the pros and cons of penile transplantation.

## Review

Methods

This article was designed as a narrative review to synthesize the existing literature on penile reconstruction and penile allotransplantation, with emphasis on surgical techniques, functional outcomes, ethical aspects, and psychosocial considerations.

A comprehensive literature search was conducted across PubMed, Scopus, Embase, and Google Scholar databases. The search covered publications from the introduction of modern microsurgical penile reconstruction techniques in the late 1990s through May 2025. Search terms and their Boolean combinations included: “penile reconstruction,” “phalloplasty,” “penile transplantation,” “vascularized composite allotransplantation (VCA),” “immunosuppression,” “psychological outcomes,” and “ethical considerations.” Reference lists of key publications were also screened to identify additional relevant studies.

Eligible studies included case reports, case series, retrospective analyses, experimental models, and review articles addressing reconstructive or transplant techniques, outcomes, immunologic challenges, and psychosocial or ethical dimensions. Formal inclusion/exclusion criteria and risk-of-bias assessments were not applied, as these are not appropriate for a narrative synthesis; instead, literature selection was guided by clinical relevance, surgical content, and publication quality.

Given the extreme rarity of penile transplantation and the limited number of reported cases worldwide (n = 5), data were synthesized qualitatively rather than through meta-analytic methods. The review emphasizes a comparative descriptive analysis across surgical, functional, and psychosocial domains.

All references were managed and formatted using EndNote v21.5 (Clarivate Analytics, London, UK).

Penile anatomy and physiology at a glance

Functional Anatomy

The penis is anatomically divided into three parts (root, body, and glans) and is composed of three erectile bodies (corpora cavernosa and corpus spongiosum). The corpora cavernosa are surrounded by a dense fibrous layer (tunica albuginea) that allows for expansion and provides rigidity during erection. The corpus spongiosum surrounds most parts of the urethra. The anatomical relationship among these four main structures of the penis varies according to the region [25]. 

These four main anatomical structures are enveloped by two fascial layers. The superficial fascia (dartos fascia) is connected to the underlying tissues by loose areolar tissue, while the deep fascia (Buck’s fascia) circumferentially invests all three erectile bodies. Arterial supply is derived from the paired common penile arteries and branches of the internal pudendal artery. Each common penile artery divides into the bulbourethral artery, cavernosal (deep penile) artery, responsible for arterial inflow during the initiation of erection, and the dorsal penile artery, which also contributes distally to the glans. These three branches form a distal anastomotic network near the glans. Arterial supply to the penile skin is provided by branches of the external pudendal arteries [26].

Venous drainage of the skin occurs via a single or paired superficial dorsal vein, which empties into one or both saphenous veins. In contrast to the paired dorsal arterial system, there is a single deep dorsal vein, which drains the corpora cavernosa via emissary veins and the corpus spongiosum via circumflex veins. Compression of these venous channels contributes to veno-occlusion during erection [26].

Penile innervation comprises both autonomic (sympathetic and parasympathetic) and somatic (sensory and motor) components. Sensory input, with accompanying sympathetic fibers, is conveyed primarily by the dorsal nerve of the penis, a terminal branch of the pudendal nerve that travels within Buck’s fascia alongside the dorsal arteries and veins. These nerves do not lie directly on the dorsal midline; rather, they course paramedian at approximately the 11- and 1-o’clock positions. Similar to the dorsal nerves, the perineal nerves arise from the pudendal nerve and supply the ventral shaft skin, the frenulum, and the bulbospongiosus muscle. The cavernous nerves originate from the pelvic (prostatic) plexus and carry parasympathetic fibers that terminate on arteriovenous anastomoses and helicine arteries of the erectile bodies; their activation promotes smooth-muscle relaxation and penile erection [26,27].

Erectile Physiology

Penile erection is a psycho-neurovascular process. Initial stimulation induces relaxation of cavernosal smooth muscle, mediated by neurotransmitters released from parasympathetic fibers (S2-S4 via the cavernous nerves from the pelvic/prostatic plexus) and by the endothelium. The key mediator is nitric oxide, which acts through the cyclic guanosine monophosphate (cGMP) second-messenger pathway. With smooth-muscle relaxation, rapid inflow through the helicine arteries fills the cavernosal spaces and produces tumescence. The ensuing distension compresses the emissary veins, limiting venous outflow; this veno-occlusive mechanism converts tumescence into a full erection [28,29].

Erection therefore depends on multiple factors: an intact psychogenic response, functional parasympathetic innervation, healthy cavernosal smooth muscle capable of relaxation, patent arterial inflow sufficient to meet demand, and an effective venous occlusive system.

Goals of penile reconstruction

The goals of penile reconstruction are to achieve a reproducible, preferably single-stage operation; to establish a competent neourethra enabling orthotopic micturition; to restore both tactile and erogenous sensibility; to provide sufficient bulk and rigidity for penetrative intercourse; to attain an aesthetically acceptable appearance with minimal scarring; and to minimize donor-site morbidity.

Table 1 summarizes the clinical indications for penile reconstruction. Phalloplasty has been performed with pedicled flaps from the thigh and inguinal regions and with various free flaps, most commonly the radial forearm, fibula, and latissimus dorsi. Nevertheless, no single technique has yet fulfilled all criteria for an ideal reconstruction [19,30].

**Table 1:** Indications for penile reconstruction

Indications for Penile Reconstruction	
Congenital	Penile hypoplasia/micropenis
	Severe hypospadias
	Severe epispadias/exstrophy–epispadias complex
Traumatic loss	Traffic accidents
	Heavy machinery injuries
	Blast injuries, gunshot wounds, and landmine explosions
	Burns
Amputations	Oncologic resection
	Self-inflicted or partner-inflicted injuries
Gender identity	Ambiguous genitalia
	Female-to-male transgender individuals

One of the major challenges in evaluating surgical outcomes and comparing different phalloplasty techniques is the lack of standardized outcome measures. Most available data come from single-institution reports, and patients’ expectations and perceptions of normal penile form and function vary widely. In particular, the priorities of individuals undergoing gender-affirming reconstruction differ from those of patients with acquired penile loss; for example, the ability to void in a standing position is generally less emphasized in gender-affirming procedures compared with reconstructive cases following trauma or oncologic resection [8].

However, the appearance of the reconstructed penis remains important in both patient groups [5]. Therefore, age, sex, and the etiology of penile loss are all key parameters in determining the most appropriate reconstructive option for each individual patient. 

Conventional methods of penile reconstruction

Since 1936, there have been various methods documented for reconstructing the penis. These conventional techniques can be categorized primarily into two groups: pedicled flaps and free flaps.

Pedicled Flap Approaches in Penile Reconstruction

Typically, a variety of abdominal flaps, including groin flaps, serve as pedicled options for penile reconstruction before the advent of microsurgical techniques in reconstructive surgery. Their primary benefit lies in providing sufficient tissue volume; nonetheless, they lack sensory function and typically necessitate a two-stage process. Moreover, the creation of the urethra using skin grafts is associated with a high rate of complications [14,17,31]. Penile prosthesis is generally required for providing rigidity to the reconstructed penis. The overall patient satisfaction is low when compared to other methods [14,17]. The anterolateral thigh flap remains the sole pedicled flap utilized in present-day penile reconstruction, with the additional capability of being employed as a free flap when required [31,32].

Microsurgical Flap Techniques in Penile Reconstruction 

Free flaps facilitate the transfer of a significant volume of tissue in a single-stage operation. Consequently, these methods gained popularity due to their capability for single-stage procedures [20,33,34]. Numerous flaps have been employed for total phalloplasty.

Radial Forearm Flap

The radial forearm flap stands out as the predominant choice for phalloplasty, boasting a notably high overall satisfaction rate (78.1%). This flap offers sensory innervation. Neo-urethra construction can be accomplished using either flap tissue or prelamination, with lower urethral complication rates compared to techniques employing skin grafts. The stiffness of the reconstructed penis can be achieved through a penile prosthesis or radial bone segment. However, the bone segment is thin and prone to fracture. The usage of prosthesis has its own drawbacks such as extrusion or infection [35-37]. Donor site morbidity including scar development and need for sacrifice of radial artery, and complications associated with penile prostheses represent the primary drawbacks of this approach [17,35,37,38].

Osteocutaneous Fibula Flap

The technique of free osteocutaneous fibular flap phalloplasty was initially introduced by Sadove and Sengezer in 1993. Subsequently, Sengezer et al. published extensive series documenting long-term outcomes in both biological male patients and transgender individuals [39-41]. Incorporating the lateral or posterior sural cutaneous nerves facilitates sensory restoration. The fibular bone segment proves effective as an autologous penile prosthesis in this method [42]. Long-term follow-ups showed minimal resorption of the fibular bone segment with sufficient stiffness for sexual intercourse [41,43]. The main disadvantage of utilizing this flap involves the necessity of employing skin grafts, harvested from the non-hear bearing skin of the groin, for urethral reconstruction [44,45]. Prolonged use of a urethral catheter for up to six months is required to prevent urethral stricture which is one of the disadvantages of the technique. To mitigate this issue, Papadopulos et al. adopted a technique involving prelamination for urethral reconstruction prior to flap transfer [46]. In spite of achieving an exceptionally high overall patient satisfaction rate, reaching 100%, the method requires a challenging surgery with broad surgical team and expertise which limits the popularity [17,47].

Scapular Flap

Scapular free flap is introduced as another alternative for total penile reconstruction, which can also be pre-expanded before tissue transfer. Scapular flap even without pre-expansion offers a large amount of soft tissue bulk for urethra and phallus reconstruction. However, to provide rigidity to the flap, a penile prosthesis is necessary, with the main drawback of the technique being the lack of a sensory nerve for restoration of erogenous sensation following penile reconstruction [17,48-50]. The latissimus dorsi flap has also been proposed for this purpose; it remains an unpopular technique. Here, thoracodorsal nerve of the latissimus dorsi muscle is coapted to recipient groin nerves to provide internal stiffness of the flap [49].

Anterolateral Thigh Flap

As mentioned earlier, this flap can serve as either a free or pedicled flap. Sensory innervation to the flap is provided by the lateral femoral cutaneous nerve. Similar to other approaches, the urethra is constructed using flap tissue, and rigidity is attained through the use of a penile prosthesis. An important benefit of this technique is the ability to achieve reconstruction with skin color matching. However, unless pre-expansion is utilized, the donor site necessitates a skin graft [13,17,32,51]. This technique offers a high satisfaction rate for total penile reconstruction [1,17].

As we discussed above, microsurgical techniques for penile reconstruction share certain similarities. Urethral reconstruction can be accomplished using either flap tissue or skin grafts. However, the neo-urethra formed by skin grafts is susceptible to high complication rates, including fistula and stricture. Conversely, the use of a penile prosthesis carries risks such as infection and extrusion. Furthermore, the penile skin and glans possess unique characteristics specific to the penis, making it impossible to achieve a naturally appearing penis using alternative tissues [5,21,35,37,52].

In the realm of penile reconstruction techniques, it is apparent that none fully align with the goals of ideal penile reconstruction. This realization is highlighted by the multitude of techniques available and the ongoing exploration for new approaches.

Penile transplantation

The breakthrough in reconstructive transplantation marks the dawn of a new era, offering solutions for cases that conventional reconstruction techniques couldn't address. Given the limited success of traditional penile reconstruction, penile transplantation emerges as a viable option for individuals with penile loss. Through this procedure, it becomes possible to reconstruct the lost corpora cavernosa, corpus spongiosum, urethra, glans, and skin using native tissues. This unique ability to replace missing anatomical components with identical ones renders penis transplantation a distinct technique capable of fulfilling the objectives of comprehensive penile reconstruction. However, it brings additional problems, which needs to be discussed in detail for each possible candidate. Although described in animal models (rats and dogs), there is limited information about the immunologic issues and erectile functions following penile allotransplantation [53-58].

To date, a total of five penis transplantations have been performed [22,59-65].

Transplantation can also enable reconstruction of adjacent tissues of penis such as scrotum or abdominal wall. Thus, the technique is called genitourinary transplantation rather than penis transplantation.

In 2006, Hu et al. performed the first human penile transplant in China on a 44-year-old male patient who lost his penis due to trauma [59,60]. Despite the successful transplantation of the pendulous part of the penis, severe psychological issues faced by the recipient and his spouse resulted in the amputation of the penis on postoperative day 14. Additionally, the patient experienced complications such as severe venous congestion and skin necrosis, further complicating the procedure.

In 2014, van der Merwe et al. conducted the second penile transplant procedure in South Africa on a patient who lost his penis during a ritual circumcision. The procedure employed microsurgical VCA techniques. Despite encountering issues with hematoma and urethrocutaneous fistula during the immediate postoperative period, the patient's long-term outcome was deemed satisfactory. One hundred days after the transplant, he regained good erectile function and a normal desire for sex. Additionally, he reported satisfactory intercourse two years post-transplantation [62,64,65]. However, the patient experienced rejection of fifty percent of the graft 32 months post-transplantation. Fortunately, this rejection was effectively treated, and the patient subsequently received skin grafts [61,65]. Furthermore, the authors reported that he successfully impregnated his partner [65].

In 2016, Cetrulo et al. carried out the third penile transplantation globally, marking the first attempt at penile transplantation in the United States [22]. The recipient had previously undergone a subtotal penectomy due to penile cancer. The surgical procedure was performed using microsurgery; the cavernosal and spongious bodies, along with the dorsal and cavernosal arteries and dorsal vein and dorsal nerves, were repaired between the donor and the recipient. Additional surgeries were necessary during the initial postoperative period, occurring on days 2 and 13 for hematoma evacuation and skin debridement due to eschar, respectively. Subsequent to these interventions, the patient reported favorable outcomes including improved urinary stream, erectile function, and sensation at the penile shaft six months post-procedure. Remarkably, even after five years following the transplantation, the patient continues to exhibit satisfactory results, with no reported issues of fistulae or strictures as reported by Lopez et al. [61].

In 2017, van der Merwe and colleagues from South Africa performed the fourth penile transplant, following their earlier successful procedure [61,65]. Subsequently, in 2021, they reported the patient's restored sexual function and his ability to engage in sexual relationships post-transplantation. Conversely, it was documented by Lopez et al. that the transplanted tissue was excised due to rejection and necrosis approximately four and a half years post-surgery [61].

In 2018, Richard J. Redett 3rd and colleagues from John Hopkins conducted the most recent (fifth) penile transplantation [63]. The recipient was a former soldier who had suffered a severe injury several years earlier due to an improvised explosive device while on duty in Afghanistan. This complex procedure involved transplanting not only the penis but also the scrotum and the lower abdominal wall. The recipient had sustained injuries across multiple areas, including severe tissue loss in the lower abdominal wall, above-knee amputation of both legs, and bilateral traumatic orchiectomy resulting in scrotal loss. The surgical team successfully transplanted the entire penis, scrotum, and lower abdominal wall. Following the procedure, the patient reported normal sensation in the penile shaft, along with the ability to achieve satisfactory erections, urinate regularly, and experience satisfactory orgasms [63].

Additional methods for penile reconstruction have been described but remain less commonly used. A comparison of the technical details of the most frequently employed procedures is provided in Table 2. Despite the breadth of techniques reported in the literature, no single approach fully satisfies the goals of ideal total penile reconstruction outlined above (see Table 3).

**Table 2:** Summary of surgical comparison of conventional penile reconstruction techniques with penile transplantation

Reconstructive Techniques		One-stage procedure	Soft tissue	Rigidity	Urethra	Sensation	Advantages	Disadvantages
Conventional Reconstruction Techniques	Radial forearm flap [35-38]	Yes	Radial forearm	Radial bone segment or penile implant	Flap tissue or prelamination	Lateral antebrachial cutaneous nerve	Reliable flap	Sacrificing the radial artery. Donor scar. Complications of the penile implant
	Osteocutaneous fibula flap [39-47]	Yes	Lateral cruris	Fibular bone segment	Skin graft or prelamination	Lateral or posterior sural cutaneous nerves	Reliable flap. No need for a penile implant	Needs expertise. Long-term usage of a urethral catheter. Permanent erection
	Scapular flap [48-50]	Yes	Lateral thoracic skin	Penile implant	Flap tissue	Not sensate	Reliable flap	Lack of sensation. Complications of the penile implant
	Anterolateral thigh flap [32,51]	Yes	Anterolateral thigh skin	Penile implant	Flap tissue	Lateral femoral cutaneous nerve	Color match. Can be used as a pedicled flap	Large donor defect requiring skin grafting. Complications of the penile implant
	Abdominal flaps [13, 14, 16]	No	Abdominal region	Penile implant	Skin graft	Not sensate	No need for microsurgery	Lack of sensation. Two-stage procedure. Complications of the penile implant
Allotransplant	Penile transplantation [22, 23, 61-65]	Yes	Penis skin	Native erectile bodies	Native urethra	Dorsal penile nerves	Possibility to restore all defective tissues	Lifelong immunosuppression. Loss of long-term results

**Table 3:** Comparison of conventional penile reconstruction and penile transplantation by outcomes

Reconstructive Techniques		Orthostatic Micturition	Sensibility	Bulk for Intercourse	Rigidity to Provide Intercourse	Aesthetically Acceptable Penis	Donor Morbidity	Overall Satisfaction
Conventional Reconstruction Techniques	Radial forearm flap [35-38]	Yes	Yes	Yes	With a penile implant	+++	+++	+++
	Osteocutaneous fibula flap [39-47]	Yes	Yes	Yes	Fibular bone	+++	++	+++
	Scapular flap [48-50]	Yes	Yes	Yes	With a penile implant	++	+	++
	Anterolateral thigh flap [32,51]	Yes	Yes	Yes	With a penile implant	+++	++	++
	Abdominal flaps [13,14,16]	Yes	No	Yes	With a penile implant	+	++	+
Allotransplant	Penile transplantation [22,23,61-65]	Yes	Yes	Yes	Native cavernous tissue	+++++	N/A	+++++

Challenges and Limitations in Penile Transplantation

Penile transplantation entails unique surgical, immunological, and psychosocial challenges. Key concerns include lifelong immunosuppression, the uncertain long-term impact of rejection episodes, and the need for rigorous patient selection and counseling.

Although offering a potential solution with native penile tissue, penile transplantation poses several challenges that must be addressed. Unlike other VCAs, many unresolved issues hinder the routine clinical application of penile transplantation. The existing literature highlights numerous areas of uncertainty surrounding penile transplantation. The removal of the penile graft in two out of the five clinical cases highlights the ongoing challenge of ensuring the long-term viability of the graft. This challenge arises from complications such as hematoma formation, vascular congestion, graft tissue necrosis, and rejection [22,61,64].

Anatomical and Technical Challenges

Although showing technical feasibility, the five cases reported in the literature also indicates technical challenges of penile transplantation. Major causes of penile loss are trauma, surgical amputation for cancer and congenital problems. The neurovascular bundle of penis includes small vessels and nerves, which may be scarred during initial trauma and cancer treatment [64]. Nerve and vessel grafts may be needed, which may affect short and long-term functional outcome. Thus, preoperative imaging may be helpful for patient selection and surgical planning [22]. Penile transplantation in the context of gender reassignment surgery presents unique challenges, particularly requiring complex dissection and meticulous surgical planning [66].

Penis has three main vascular perfusion zones. Briefly, glans and spongiosa body are perfused by dorsal penile arteries. Cavernosal bodies are supplied by cavernosal arteries. These vessels originate from internal pudendal arteries. The penile shaft skin, on the other hand, is supplied from external pudendal system. During penis transplantation, all these arteries needed to be anastomosed for sufficient perfusion and erection [67]. Anastomoses of cavernosal arteries are accepted to be very important for adequate erection at the postoperative period, although penile replantation data shows controversy reports about the role of cavernosal artery anastomosis [68].

Urethral and Erectile Function-Related Challenges

Two fundamental roles of the penis include urination and achieving an erection. There is a lack of clinically translational animal models of functional penis transplantation, and we have a limited number of clinical cases. Data on long-term functional results are insufficient [65]. So, to some extent, we may use penile replantation outcomes to predict the functional outcomes of penile transplantation. Urethral stricture poses a significant challenge in penis reconstruction procedures [13,17]. The incidence of urethral complications following penile replantation and reconstruction ranges from approximately 6.7% to as high as 68% [14,69-71]. However, in transplantation settings, the long-term effects of acute or chronic rejection episodes on urethral tissue remain uncertain. It is conceivable, however, that these conditions could potentially induce fibrosis and lead to the development of severe urethral strictures over time.

Morrison et al. evaluated and recently reported a retrospective cohort analysis about complications and functional outcomes of penile replantation [72]. They included 107 penile replantation cases. Full sensation and diminished sensation were reported in 68.4% and 28.6% of cases, respectively. Urinary function was satisfactory in 97.4% of patients; however, normal erection was reported in only 77.5 % of the patients. Thus, it is evident that both limited clinical data and penile replantation literature shows that, penile transplantation offers a functional penis regarding erection, urinary function, and sensation. However, these cases include replantation or transplantation of the pendulous part of penis, which has intact recipient vasculature. Thus, our knowledge and data are based on patients with intact proximal corporal stumps which preserve tumescence. When the proximal cavernous tissues are injured or not present, functional erection may not be obtained requiring penile injections with vasoactive substances or a penile prosthesis. If the proximal cavernous tissues are damaged or absent, achieving a functional spontaneous erection may be unattainable. The lack of appropriate animal models poses a significant hurdle in both understanding and addressing potential erectile dysfunction post-penile transplantation [53].

The initial outcomes of penile transplantation show promising results regarding erectile function. However, the long-term sustainability of erectile function in transplanted penises remains uncertain [23,61,73]. Based on available data, the long-term use of immunosuppressive agents may lead to a range of adverse effects, including cancer, infections, risk of multiple episodes of rejection, metabolic disorders, and even mortality [74]. These agents can significantly impact the quality of life, with potential side effects such as diabetes mellitus affecting erectile function [75]. Issues arising from immunosuppression, such as diabetes mellitus, or chronic rejection impacting the vascular system, can lead to secondary impotence, potentially necessitating medical, stem-cell therapy, or surgical interventions for resolution. However, surgical interventions, such as the insertion of a prosthetic device, carry additional risks of infection and rejection. Despite successful treatment of these episodes, the impact on erectile function remains unknown. Therefore, careful consideration is needed to balance the benefits of allograft protection against the risks of postoperative immunosuppressive therapy. Currently, there are no established guidelines for the use of immunosuppressive agents in penile transplantations, highlighting the need for further research to address this challenge.

Organ Donation Challenges

Solid organ donation is more prevalent than VCA donation, families may find it easier to consent to solid organ donation compared to vascularized composite tissue donation. Considering factors such as marital status, religious beliefs, psychological aspects, social implications, and individual identity, the decision to donate the penis could pose greater challenges for the family [71,76-79]. Considering donor site-related concerns, it's notable that the pubic area, unlike hand or face VCAs, remains unexposed during funeral ceremonies. This presents a significant advantage, eliminating the need for surgical teams to replace it with silicone products [71].

Immunology-Related Challenges

Immunology-related challenges represent the primary limitations of all VCAs, and likewise, penile transplantation necessitates lifelong immunosuppression to mitigate acute and chronic rejection. Like other VCAs, penile transplant patients require chronic, systemic, non-specific immunosuppression which brings too many adverse effects [80,81]. The ethical concerns also focus on this issue as penile transplantation is not lifesaving procedure. Thus, the optimal immunosuppressive regimen remains imprecise for many VCA procedures, including penile transplantation. There is sufficient evidence to suggest that the immunosuppressive regimen could influence the long-term functional outcomes of the transplanted penis [82]. A potential advantage could lie in the utilization of topical immunosuppressive medications for the management of penile transplants, particularly those involving skin and mucosa [83].

During the clinical monitoring of VCAs, it is crucial to promptly identify potential rejection episodes, necessitating routine or selective skin biopsies for histological assessment and grading of rejection [84]. The antigenic burden associated with penile transplantation is anticipated to be lower, because penis carries small amount of skin and other tissues in contrast to the hand and face, and this may be an advantage in terms of immunosuppression need [80,85]. On the other hand, this advantage also disables routine or for cause biopsies for immune monitoring of the transplanted skin. Although low antigenic load, the third case of penis transplantation experienced steroid-resistant acute rejection episode which needed a repeat course of methylprednisolone and anti-thymocyte globulin [22]. Moreover, it should be noted that performing skin biopsies may provoke rejection episodes and exacerbate harm to the allograft. Hence, sentinel flaps might be necessary for monitoring the immunological condition of the allograft [86]. Moreover, the grading and manifestation of rejection could vary from other VCAs since the skin of the penile shaft is hairless, highly elastic, and devoid of subcuticular fat, distinguishing it from other anatomical regions. Thus, there is an unmet need for noninvasive monitoring of the penile skin for acute rejection episodes.

Certain immunosuppressive medications, like Cyclosporine A, have been linked to an increased risk of erectile dysfunction [82]. However, due to the limited number of cases conducted worldwide, it is challenging to make conclusive determinations regarding the most suitable immunosuppressive regimen. Therefore, the ideal immunosuppression protocol for penile transplantation may differ from that employed for hand or face transplants. In their ex vivo study of human penile transplantation and rejection, Sopko et al. observed that FK506 might be a more suitable immunosuppressant for penile transplantation [87].

The issue of chronic rejection in VCA, which can result in vascular obliteration and eventual graft failure during long-term follow-up, presents significant challenges for penile transplantation [88,89]. Even if the penile flap remains viable, chronic rejection may lead to erectile dysfunction in the long run. Additionally, repetitive trauma in VCA is a crucial mechanism for triggering rejection episodes, and repeated trauma from the recipient's sexual activity may pose a risk factor for acute rejection [54].

The increased frequency of early wound healing complications subsequent to penile transplantation poses an additional obstacle to the procedure. It's conceivable that perioperative wound healing complications and subsequent surgical interventions aimed at resolving these issues could induce inflammation, thus activating innate immunity. Despite the administration of induction therapy, inflammation might lead to allorecognition, presenting further immunological hurdles to penile transplantation [90]. In instances of chronic rejection, the transplanted penis may necessitate amputation, mirroring occurrences observed in hand transplantation. Furthermore, despite adequate penile circulation, chronic rejection could adversely impact the smaller vessels, affecting erectile function [90].

For patients undergoing face transplantation, the process of implementing rescue procedures and transitioning to subsequent reconstruction steps is more complex compared to those undergoing hand transplantation. However, for penile transplant recipients, these aforementioned issues parallel those encountered in hand transplants. Similar to hand transplantation scenarios, the removal of a penile allograft can be readily performed if the viability of VCA is jeopardized or if life-threatening circumstances arise. Patients should be duly informed that the allograft may be excised if the balance between benefits and risks becomes unfavorable [71].

Ethical and psychosocial considerations

Ethical Challenges in Genitourinary Transplantation

Ethical and psychosocial factors play a central role in candidate selection, consent, and graft retention. Partner involvement, recipient acceptance, and realistic expectation management are critical determinants of outcomes.

The ethical controversy related to penis transplantation is perhaps the most important challenge. As mentioned above, penis transplantation is not a lifesaving, but rather life-enhancing procedure and the risk/benefit ratio must be assessed accordingly. The primary ethical concern regarding penis transplantation is whether the potential improvement in quality of life justifies the well-established risks associated with long-term immunosuppression [53,91]. Hand and face transplantations are performed to patients who cannot be reconstructed by conventional methods. However, there are several methods of total penile reconstruction with acceptable long-term results [17,41]. Therefore, patient selection becomes very important for determining potential candidates of penis transplantation.

Psychological and Partner-Related Factors

Unlike solid organ grafts, penis and other VCA grafts are visible and have marked implication on the recipient's sense of self. The process of adapting to a new graft involves integrating into the recipient’s sense of identity and bodily integrity. Failure to adapt psychologically and emotionally to the graft is associated with adverse clinical outcomes, as seen in the first penis transplant. When considering penile transplantation, the impact on sexuality and implications for sense of identity and sexual intimacy in the context of intimate partner relationships are specific to this procedure and, thus, warrant careful consideration and study [53].

Patients undergoing penile transplantation face significant psychological hurdles both before and after the procedure. Pre-transplantation, these challenges revolve around the emotional aspect of living with a penile defect, such as amputation, which can cause profound distress. Merwe et al., who conducted two penile transplantation cases, highlighted the immense psychological impact of penile loss, emphasizing its devastating effects on self-esteem and identity. Both patients expressed severe depression, occasionally contemplating suicide, feeling ostracized, worthless, and ashamed. Following transplantation, a range of psychological issues emerges due to the distinctive aspects of penile transplantation [65]. The recipient's psychological readiness to accept the donor's penis is essential, necessitating readiness from both the recipient and their partner [53,61,71,73,91,92]. It is crucial to evaluate the mental health of the recipient's partner both before and after the surgery, as their acceptance of the graft is equally crucial to the success of the procedure.

The first reported penile transplantation, performed by Zhang et al., necessitated graft removal within 14 days, underscoring the profound impact of psychological distress in both the recipient and their partner as a critical barrier to graft retention [91]. This early experience underscores that psychosocial stability and partner acceptance are prerequisites for graft retention.

In a subsequent review, the same group proposed guiding principles for penile transplantation, emphasizing ethical considerations, psychological factors, and stringent patient selection, thereby highlighting psychosocial stability as a central determinant of long-term success [59,60,91].

Balancing Quality of Life and Medical Risk

Although the procedure's efficacy and safety are being evaluated, clear indications for transplantation and comprehensive ethical frameworks are necessary. To address these issues, Ngaage et al. proposed guidelines called "The Baltimore Criteria for an Ethical Approach to Penile Transplantation" after conducting a literature review and drawing from their own experience [93]. This type of research can educate involved parties about relevant principles and assist in creating protocols for selecting and prioritizing candidates [52,93].

Distinct from solid organ transplants, VCAs like penile transplantation do not save lives but rather enhance them. The ethical landscape of transplantation has evolved with the advent of reconstructive procedures [94] . While VCA can enhance quality of life, it may come with the trade-off of potentially shortening life expectancy, prompting ethical debates on whether the benefits justify the risks. It is crucial to clearly establish the expectations of individuals who have undergone penile loss. Contrary to this belief, Patel (2018) indicated that penile transplantation does not possess qualities that are either life-saving or capable of enhancing quality of life when compared to the existing alternative, phalloplasty [95]. Consequently, he argued that penile transplantation cannot be deemed a medical priority, as the potential advantages are not deemed to outweigh the associated risks and expenses. Therefore, there is a critical need for objective assessments of both the functional and aesthetic outcomes of traditional techniques. VCA grafts are visible and exert a significant impact on the recipient's sense of identity [52,96,97]. Moreover, the penis holds substantial importance in sexuality and intimate relationships, adding layers of complexity to the ethical considerations surrounding penile transplantation.

The challenges associated with penile transplantation highlight the need for new, clinically relevant animal models and extensive clinical research to understand the effects of transplantation on penile function. Thus, meticulous patient selection is crucial in identifying potential candidates for penis transplantation in clinical studies. While there are numerous articles discussing patient selection, consensus on recipient selection criteria is still lacking.

Penile reconstruction versus transplantation

From a reconstructive standpoint, penile transplantation fulfills nearly all the objectives of an ideal penile reconstruction. It offers a single-stage procedure that is reproducible, creates a competent urethra, and results in a sensate, aesthetically normal, and erectile penis without donor site morbidity. Transplantation also enables the simultaneous reconstruction of adjacent structures, such as the scrotum or abdominal wall, making whole genitourinary transplantation feasible [5,17].

Despite these advantages, penile transplantation remains limited by several major challenges. The foremost are immunological and ethical concerns, compounded by the absence of long-term outcome data. The lifelong requirement for immunosuppression inevitably reduces life expectancy, with risks including malignancy and fatal organ failure, while acute or chronic rejection episodes may compromise function, potentially leading to erectile dysfunction.

By contrast, conventional reconstruction, though imperfect, carries no risk of immunosuppression and preserves life expectancy. In comparison, conventional reconstructive techniques although capable of providing patients with a penis generally considered functional and satisfactory are often associated with higher rates of urethral complications and do not restore natural erectile function or a normal appearance.

Thus, comparison between the two techniques becomes a question of quality of life versus quantity of life [94]. Penile reconstruction provides an acceptable outcome compatible with normal survival, whereas transplantation offers the possibility of a natural-appearing and functional penis but at the cost of diminished life expectancy. Given the limited number of reported cases and the short duration of follow-up, decisions must be individualized, weighing risks and benefits on a case-by-case basis. Importantly, ongoing advances in immunosuppressive therapies and tolerance-inducing strategies will be critical in shaping the future role of penile transplantation. To facilitate clarity and organization, key information has been summarized across three comparative tables: Table 1 (indications), Table 2 (surgical comparison), and Table 3 (outcomes' comparison). A technique-level synopsis is provided in Table 2, and an outcome-oriented comparison is summarized in Table 3. To facilitate direct comparison, the key distinctions between conventional penile reconstruction and penile allotransplantation are summarized in Table 4.

**Table 4:** Summary of differences between conventional penile reconstruction and penile allotransplantation* RFF, radial forearm flap; ALT, anterolateral thigh; VCA, vascularized composite allotransplantation; IS, immunosuppression; FTSG, full thickness skin graft *This summary table synthesizes key features discussed in the manuscript; see the text for citations.

Feature	Conventional Penile Reconstruction	Penile Allotransplantation
Tissue source	Autologous flap (e.g., RFF, ALT)	Donor allograft (VCA)
Urethra	Flap-based/FTSG neourethra construction	Native urethral continuity
Sensibility	Variable; depends on nerve coaptation	Potential for near-native sensibility with nerve integration
Rigidity	Prosthesis or autologous support	Native corporal bodies
Aesthetics	Variable; flap-dependent	Anatomically natural appearance
Donor-site morbidity	Present at flap harvest site	None for recipient
Immunosuppression	Not required	Lifelong immunosuppression required
Complication profile	Urethral stricture/fistula, prosthesis issues	Rejection risk, infection, IS-related adverse effects
Functional outcomes	Orthotopic voiding feasible; sexual function variable	Potential for near-physiologic urinary and sexual function
Psychosocial considerations	Prosthesis dependence and appearance may influence body image and satisfaction.	Identity integration and body image may improve, although psychological adjustment can be complex
Ethical/regulatory	Minimal	Donor allocation and consent/ethics considerations
Follow-up/monitoring	Standard reconstructive follow-up	Graft surveillance and immunologic monitoring
Chronic rejection / long-term function	No immune-mediated rejection; however late complications (e.g., urethral issues, prosthesis failure, limited sensory return) may impair erectile function	Risk of chronic rejection and transplant vasculopathy with potential late decline in erectile, urinary, or sensory function; lifelong immunologic surveillance required
Cost/Accessibility	Generally, more accessible; costs tied to flap surgery, prosthesis, and revisions	Limited to few centers; ongoing IS, rejection workup, and high-acuity follow-up increase cost
Learning curve/center experience	Extensive reconstructive experience; outcomes improve with surgical experience and case volume	Highly specialized VCA programs; outcomes depend on multidisciplinary transplant expertise

Future perspectives

There is no doubt that as in all fields of transplantation, immunologic developments including safer drugs and creation of a method for donor-specific tolerance will affect the penis transplantation. In terms of penile transplantation, there are still many unknowns, and additional studies are needed to clarify these issues. One of the important points is lack of preclinical data about penis transplantation. A clinically relevant animal model is needed to evaluate the immunologic and functional challenges. We also need additional clinical cases with longer follow up times.

Testis transplantation is another issue that can be considered with penis transplantation in the future. Testicle transplantation could enable men without these sex organs (due to congenital or acquired absence) to conceive naturally without the use assisted reproductive technology. Testis transplantation is first described between identical twin brothers by Silber in 1978, and the transplantation was successful with the infertile brother having five children [98]. Although there is no doubt that this will bring additional ethical arguments, there is an increasing demand for reproductive tissue transplants (transplantation of ovaries, uteruses, and testicles), which present new opportunities for patients with a variety of fertility conditions.

Another developing area, which may be applied to transplantation, is regenerative medicine. Advances in regenerative medicine and tissue engineering may help penis reconstruction to new frontiers [99]. Tissue engineering strategies may be combined with reconstructive and transplantation methods. Currently, there are increasing clinical studies about using acellular and recellularized tissue-engineered urethral constructs in urethral reconstruction [100]. There are continuing studies aimed at tissue engineering of penis [101].

## Conclusions

Conventional reconstructive techniques can achieve acceptable and functional results, but they remain imperfect, with high complication rates and limited ability to restore a natural, aesthetically normal, and erectile penis. Penile transplantation has emerged as a novel VCA procedure that offers the potential for definitive treatment in selected patients with severe penile disfigurement, addressing both functional and psychosocial concerns. However, transplantation is accompanied by substantial challenges, including the risks of lifelong systemic immunosuppression, the uncertainty of long-term outcomes, and the ethical and psychosocial complexities surrounding donor and recipient considerations.

At present, penile transplantation remains experimental, with very few cases and limited follow-up data. Critical issues such as candidate selection, donor perspectives, the impact of acute and chronic rejection on functional outcomes, and long-term complications including malignancy and organ failure related to immunosuppression require further investigation. Psychosocial stability, patient acceptance, and partner support have also been shown to be decisive in determining graft survival. Progress in this field will depend not only on continued clinical experience but also on the development of robust preclinical models to refine surgical techniques, understand immunological mechanisms, and evaluate long-term functional outcomes. Until these questions are answered, penile transplantation should be approached cautiously and considered only in highly selected cases where conventional reconstruction cannot provide an acceptable result.
